# Panax ginseng C.A Meyer root extract for moderate Chronic Obstructive Pulmonary Disease (COPD): study protocol for a randomised controlled trial

**DOI:** 10.1186/1745-6215-12-164

**Published:** 2011-06-30

**Authors:** Charlie C Xue, Johannah L Shergis, Anthony L Zhang, Christopher Worsnop, Harry Fong, David Story, Cliff Da Costa, Francis CK Thien

**Affiliations:** 1 Discipline of Chinese Medicine, School of Health Sciences and Health Innovations Research Institute (HIRi), RMIT University, Bundoora, VIC, Australia; 2 Department of Respiratory and Sleep Medicine, Austin Hospital, Heidelberg, VIC 3081, Australia; 3 Department of Medicinal Chemistry and Pharmacognosy, College of Pharmacy, University of Illinois at Chicago, IL 60612, USA; 4 School of Mathematical and Geospatial Sciences, RMIT University, Bundoora, VIC, Australia; 5 Department of Respiratory Medicine, Box Hill Hospital and Monash University, Box Hill, VIC 3128, Australia

## Abstract

**Background:**

Chronic obstructive pulmonary disease (COPD) impairs quality of life and leads to premature mortality. COPD sufferers experience progressive deterioration of lung function and decreased ability to undertake day-to-day activities. Ginseng has been used for thousands of years in Chinese medicine for respiratory symptoms. Several controlled clinical trials using ginseng for COPD have shown promising clinical effect, however these studies were generally small and with some potential bias, prompting the need for rigorously designed studies.

**Aim:**

The objective of this study is to evaluate the therapeutic value and safety profile of a standardised root extract of Panax ginseng C.A Meyer (ginseng) for symptomatic relief, with a focus on quality of life (QoL) improvements in individuals with moderate (Stage II) COPD FEV1/FVC < 0.7 and FEV_1_ 50% - 80% predicted.

**Methods:**

This paper describes the design of a randomised, multi-centre, double-blind, placebo controlled, two-armed parallel clinical trial. Two trial sites in Melbourne Australia will proportionately randomise a total of 168 participants to receive either ginseng capsule (100 mg) or matching placebo twice daily for 24 weeks. The primary outcomes will be based on three validated QoL questionnaires, St Georges Respiratory Questionnaire (SGRQ), Short Form Health Survey (SF-36) and the COPD Assessment Test (CAT). Secondary outcomes are based on lung function testing, relief medication usage and exacerbation frequency and severity. Safety endpoints include blood tests and adverse event reporting. Intention-to-treat will be applied to all data analyses.

**Discussion:**

Findings from this study may lead to new therapeutic development for chronic respiratory diseases, particularly COPD. This protocol may also guide other investigators to develop quality herbal medicine clinical trials in the future.

**Trial registration:**

Australia and New Zealand Clinical Trials Register (ANZCTR): ACTRN12610000768099

## Background

Chronic obstructive pulmonary disease (COPD) is a major cause of morbidity and mortality worldwide [1]. Current estimates of its prevalence in the adult population (≥ 40 years old) are reported at approximately 10% [2] with global leading cause of death projections ranking COPD 3^rd ^by 2030 [3]. COPD is widely accepted as a preventable disease, but due to continued inhalation of noxious substances, particularly cigarette smoke, COPD is expected to be a major burden for individuals and health care providers for the foreseeable future. Due to limited reversibility and progression, mild symptoms can develop in severity particularly if smoking persists and symptoms are poorly managed. Even after smoking cessation individuals can experience progressive airflow limitation and increased shortness of breath, chronic cough, sputum production and impaired quality of life (QoL). Therefore, early diagnosis and treatment of COPD can reduce the likelihood of symptom deterioration, disease progression and improve overall outcomes [4].

Severity classification of COPD has been defined by the Global Initiative for Chronic Obstructive Lung Disease (GOLD) into four stages, mild (Stage I), moderate (Stage II), severe (Stage III) and very severe (Stage IV). Classification is determined by spirometric measurements using post-bronchodilator forced expiratory volume in 1 sec (FEV_1_) and its ratio to forced vital capacity (FVC) [5]. All stages of COPD, mild to very severe are associated with impaired QoL [6].

Currently there is no cure for COPD. Due to its irreversible nature, conventional treatments primarily intend to control and alleviate symptoms, and to prevent complications. Commonly used treatments include bronchodilators, corticosteroids (for severe cases), and antibiotics (for exacerbations). However, these treatments produce only modest increases in lung function and are associated with a range of adverse effects [7,8]. With improved understanding of the cellular and molecular mechanisms involved in COPD, some novel approaches to treatment are currently under investigation, including pro-inflammatory mediator antagonists, protease inhibitors and phosphodiesterase 4 (PDE4) inhibitors [8]. Yet, for the drugs so far developed, clinical usefulness has been limited by adverse effects [8,9].

Current internationally accepted guidelines recommend symptomatic treatment for COPD with short or long-acting bronchodilators [5]. The addition of regular treatment with inhaled steroids to bronchodilators is only appropriate for more severe symptomatic patients FEV_1 _< 50% predicted (GOLD stages III and IV) and with repeated exacerbations [1]. Although commonly used in mild to moderate COPD patients (GOLD stages I and II) in the community, inhaled steroids are not recommended for this group of patients [5]. It is in this group of symptomatic moderate patients (GOLD stage II), who are often exposed to inhaled steroids without any evidence of benefit, but are at risk of long-term side-effects such as cataracts and osteoporosis, that we seek an alternative and safe treatment approach in this important clinical study.

There is growing interest and use of complementary and alternative medicines for the management of COPD. A recent study in Australia suggested that nearly one in six (17.3%) individuals with COPD had used some form of herbal preparation [10]. Despite increasing use by COPD sufferers, there is inadequate evidence to support routine use of herbal therapies.

Panax ginseng C.A Meyer (ginseng) root is an important Chinese herbal medicine in use for thousands of years. Recently pharmacologically active constituents have been identified, most notably ginsenosides [11]. Potentially relevant activities of these constituents for COPD include, inhibition of pro-inflammatory mediators and cytokines [12], reduction of oxidative stress [13], anti-protease properties [14] and elevation of cAMP [15]. As well as having pharmacologically active constituents, ginseng has an excellent safety profile and is well tolerated by patients [16].

A recent systematic review evaluating ginseng formulae for stable COPD showed promising evidence of lung function and QoL improvements. Included studies had certain methodological weaknesses [17] however these encouraging results prompt the need for rigorously designed randomised controlled trials (RCTs) of high methodological quality.

## Objective

The objective of this study is to evaluate the therapeutic value and safety profile of a standardised root extract of Panax ginseng C.A Meyer (ginseng) for symptomatic relief, with a focus on QoL improvement in individuals with moderate (GOLD Stage II) COPD (FEV_1_/FVC < 0.7 and FEV_1 _50% - 80% predicted). To fulfil the objective, answers to the following questions will be addressed 1) Does a standardised extract of ginseng produce beneficial effects/symptomatic relief in subjects with moderate COPD, in terms of improved QoL and/or improved respiratory function? 2) Does standardised ginseng extract reduce the use of conventional symptom-relief medication in subjects with moderate COPD? 3) With six months treatment and six months follow-up, what are the time courses of onset and persistence of any beneficial effects of the ginseng extract in subjects with moderate COPD? 4) What adverse events does the ginseng extract produce in subjects with moderate COPD?

## Methods

### Design

This study is a randomised, multi-centre, double-blind, placebo-controlled, two-armed parallel clinical trial, comparing a standardised ginseng extract to placebo. The design of the study will integrate rigorous, contemporary clinical research methodology in accord with principles set out in the Declaration of Helsinki and the Good Clinical Practice guidelines with the theory that guides the appropriate use of traditional Chinese medicine in clinical practice. Reporting will be guided by the CONSORT statement [18,19], including relevant intervention extensions relating to herbal medicines [20].

**Figure 1:**
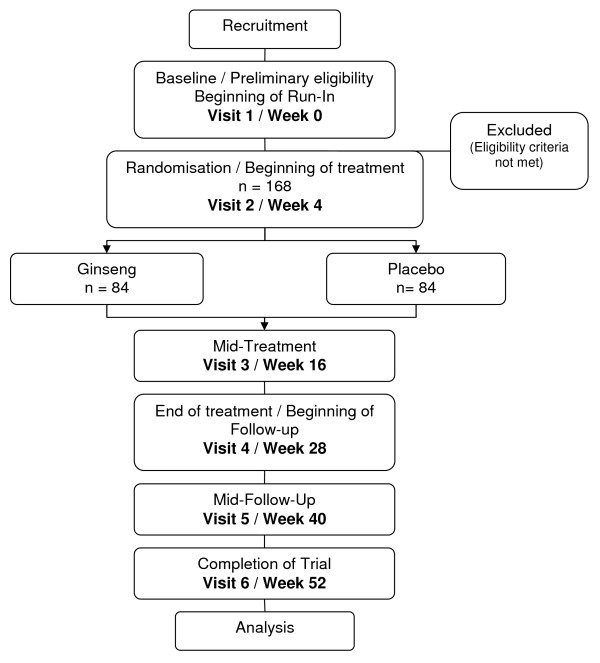
**Participation Flow Chart**.

Two hospitals in Melbourne, Australia will randomly assign 168 patients with moderate COPD at a ratio of 1:1 into the ginseng group or matching placebo. Consenting eligible participants will be enrolled for 52 weeks and will be required to attend 6 visits in total. The trial is designed in three phases, 4 weeks run-in; to ensure participants are clinically stable with respect to their COPD, 24 weeks treatment; with either ginseng or placebo capsules, and 24 weeks follow-up (Figure 1).

### Study Duration

Ongoing recruitment will occur for a maximum period of 21 months (October 2010 and June 2012) or until a sample of 168 individuals are randomised.

### Subjects

168 participants will be recruited through local advertising and doctor referrals from hospital outpatients and general practice clinics. Interested participants can telephone or email the trial co-ordinators at the corresponding sites for further information. Participant information and consent forms will be sent to interested individuals to read over prior to scheduling their first visit.

#### Inclusion criteria for participation in the trial

1) males and females aged 40 to 80 years inclusive; 2) ex-smokers (ceased smoking at least three months prior trial entry), and agree to refrain from smoking throughout the trial; 3) satisfy the COPD diagnostic criteria for moderate (stage II) defined by GOLD as post-bronchodilator spirometry, FEV_1_/FVC < 0.7 and FEV_1_ 50% - 80% predicted; 4) are clinically stable, that is, did not experience an acute infective exacerbation of COPD from at least 4 weeks prior to trial entry; 5) meet the Chinese medicine diagnostic criteria for Lung Qi deficiency or Lung and Spleen Qi deficiency; and 6) give written informed consent to participate.

#### Exclusion criteria

1) current smokers; 2) individuals with a diagnosis of alpha_1_-antitrypsin deficiency; 3) a history of asthma or chronic systemic infections or inflammatory conditions in the last three months; 4) pregnancy, breast-feeding or women intending to become pregnant during the course of the study; 5) serious illnesses such as heart, liver or kidney diseases; 6) those who are unable to adequately perform spirometry tests; 7) those taking long-term immunosuppressive agents or immunostimulants; 8) those who have an allergic history to ginseng products; 9) those currently using a ginseng-containing product or have used a ginseng product within the last three months; 10) those who are current users of anticoagulants, anti-hyperglycaemics or monoamine oxidase inhibitor anti-depressants; and 11) those who have undertaken pulmonary rehabilitation within three months of the commencement of the study, or intend to enter pulmonary rehabilitation during the study.

Preliminary screening for eligibility will be undertaken at Visit 1 and during the 4-week run-in period. After this period if suitability continues, participants will be randomly assigned to one of the two treatment groups, ginseng or matching placebo. Continued suitability requires that they be clinically stable and not have experienced an acute infective exacerbation of COPD during the run-in period and that post-bronchodilator spirometry values stay within the moderate (Stage II) range. Individuals who do not meet the preliminary criteria will not be randomised and their participation in the trial will be discontinued. Participants' self-reported respiratory symptoms, smoking status, current medications and other medical conditions will be documented at baseline and throughout.

As well as a conventional diagnosis of COPD performed by a physician, participants must also meet the appropriate Chinese medicine syndrome classifications of Lung Qi deficiency or Lung and Spleen Qi deficiency. A registered Chinese medicine practitioner will perform this diagnosis and only those with either of the two syndromes will be accepted into the study.

### Ethics

This study has been approved by the relevant local human research ethics committees (HRECs) of the participating centres. Any amendments to the study protocol will be submitted to the HRECs for approval. HREC reference numbers: Austin Health HREC/10/Austin/8 (H2010/03892), Eastern Health E90/0910 and RMIT University E31/10.

Clinical trial notification has been filed with the Therapeutic Goods Administration (TGA) Australia (CTN numbers 2010/0449 for Austin Health and 2010/0419 for Eastern Health).

### Randomisation

All eligible subjects will be randomised using two different sizes of block randomisation sequences generated by computer and stratified by site. Treatment allocation numbers will be entered into individually sealed opaque envelopes and provided to each site. At the time of randomisation participants will draw an envelope. Each envelope contains a number that is concealed to the treatment allocation. Randomisation sequence and allocation will be concealed to all study subjects, research staff, investigators and pharmacists until completion of the study. The allocation list will be protected by password access files and held by a non-investigator independent. In the event of an emergency medical situation the individual's randomisation code and group allocation could be identified.

### Sample size

The sample size calculation is based on the effect size on QoL changes in COPD subjects, in a RCT of one of the most commonly used long-acting beta_2_-adrenoceptor agonists (salmeterol) [21]. In the study, the mean score change on the St Georges Respiratory Questionnaire (SGRQ) was significantly higher (*p *< 0.05) in the treatment group (-6.8 ± 13.2) than in the placebo group (-1.4 ± 11.7) [21]. For the proposed study, to achieve a similar difference between the ginseng extract and placebo treatment groups with an 80% power and a two-tailed significance level of 5%, it is estimated that a sample size of 84 subjects per group will be required, that is, 168 in total. Intention-to-treat analysis will be applied to minimise bias due to drop-outs.

### Treatment

A standardised Panax ginseng C.A Meyer (Araliaceae; Asian ginseng or *Renshen*) root extract will be used as treatment. The ginseng and matching placebo (i.e. identical appearance, taste and odour) will be dispensed as 100 mg gel-filled soft capsules for oral intake twice daily, for a total of 24 weeks. The standardised ginseng extract and matching lactose-based placebo will be manufactured in accordance with good manufacturing practice (GMP) by Ginsana SA, Switzerland. The ginseng extract will be standardised to contain 4% ginsenosides. Routine quality control checks will be undertaken on the packaging and contents of the batch to ensure stability and quality with handling and storage also following GMP procedures. The dosage of ginseng was determined by referencing previous clinical trials for the same indication [22,23], and by recommendations from the manufacturer. This product is listed for use as an herbal medicine with the TGA Australia (ARTG 14987).

Throughout the trial, participants will be supplied with symptomatic relief medication, a short acting beta2-agonist Salbutamol, to be used on an as needed basis. To monitor medication compliance, participants will be required to document such use in a take home diary. Participants will be asked to return any capsules they have neglected to take so that these can be checked against the diary entries.

Participants will be advised not to take certain COPD medications throughout the trial. Such as; short acting anti-cholinergics, long acting beta2-agonists, theophylline, inhaled corticosteroids (ICS) and combination ICS/beta2-agonists. ICS are recommended for more advanced COPD sufferers and those with repeated exacerbations [1]. The use of a long acting anti-cholinergics will be allowed.

If exacerbations occur, management will be decided by the patients' own physician/healthcare provider and all relevant details including medications will be recorded by the research investigators as secondary outcomes. The trial defines exacerbations as at least a 2-day persistence of at least two major symptoms, such as worsening dyspnoea and an increase in sputum purulence, volume, or both, or of any single major symptom plus more than one minor symptom (upper airway infection, unexplained fever, increased wheezing) [24].

### Outcomes

The primary outcome measures will be QoL improvement assessed by using three validated and self-administered questionnaires.

• St Georges Respiratory Questionnaire (SGRQ)

• Short Form Health Survey (SF-36); and

• COPD Assessment Test (CAT)

Participants will complete the three questionnaires at each visit, 6 times in total (Week 0, 4, 16, 28, 40 and 52). (Table 1)

**Table 1:** Measurements to be taken throughout the trial

Visit/Week	1/0	2/4	3/16	4/28	5/40	6/52
**SGRQ**	✓	✓	✓	✓	✓	✓

**SF-36**	✓	✓	✓	✓	✓	✓

**CAT**	✓	✓	✓	✓	✓	✓

**Spirometry**	✓	✓	✓	✓	✓	✓

**Full Blood Count**	✓(Including alpha_1 _antitrypsin)			✓		

**Blood Biochemistry** **KI & LR function**	✓			✓		

CAT: COPD Assessment Test; KI: Kidney; LR: Liver; SF-36: Short Form Health Survey; SGRQ: St Georges Respiratory Questionnaire.

Secondary outcome measures include efficacy and safety components.

Lung function using spirometry indices of FEV_1 _and FEV_1_/FVC ratio.

Use of relief medication short-acting bronchodilator (beta2-agonist Salbutamol)

Frequency, nature and severity of exacerbations

Emergency department presentations and medical practitioner visits

Full blood examination and blood biochemistry for liver and renal function

Adverse event reporting

Spirometry at each site will be conducted using SpiroUSB™ (CareFusion) and Spida5^® ^software. Routine calibration and standard operating procedures will be uniformly undertaken at each site. Participants will be asked to perform spirometry at each visit, 6 times in total (Week 0, 4, 16, 28, 40 and 52). Participants will be advised not to use respiratory medications for several hours prior to their spirometry testing. Two sets of measurements will be taken at each visit, pre-bronchodilator and post-bronchodilator (post 400 μg of inhaled Salbutamol).

Any adverse events will be listed in the trial record and followed-up to completion by the trial co-ordinators and respiratory physicians. Adverse event details will be scored using a six-point scale, 0 = none, 1 = minimal, 2 = mild, 3 = moderate, 4 = severe, and 5 = extremely severe. Participants will be able to report adverse events anytime throughout the trial and will receive advice accordingly. Serious adverse events will be reported to the reviewing HREC and site HRECs within the timeframe specified by the lead HREC.

Throughout the study period (52 weeks) trial coordinators will contact the participant's usual treating doctor to record relevant data on presentations and exacerbations.

To assist with outcome documentation and medication compliance, participants will be given a participant diary to complete on a daily basis for the duration of the trial. They will be asked to record trial medication and relief medication usage as well as any new medications they commence during the trial.

Participants may withdraw from the study for any reason at any time without repercussion. They will only be withdrawn by investigators if it is deemed medically unsafe for them to continue. Dropouts will not be replaced.

### Statistical analysis

The trial data will be analysed by an independent biostatistician who will be blinded to the subject allocation. Primary outcome measures SGRQ, SF-36 and CAT scores of participants in the treatment and placebo groups will be assessed at baseline, start of treatment (4 weeks) mid-treatment (16 weeks), end of treatment (28 weeks), mid follow-up (40 weeks) and at the end of follow-up (52 weeks). A suitable statistical package such as SAS will be used to analyse the data. An intention-to-treat analysis will be applied using the Last Observation Carried Forward (LOCF) method. Analysis of covariance with baseline as covariate will be used to assess differences in treatment outcomes between the two groups at each of these time points. To correct for inflated risk of Type 1 error, multiple comparison procedures suggested by Ludbrook et al will be used [25,26]. The use of relief medication during the trial will be investigated for its effect on the outcome measures by using it as a covariate in the statistical analysis. A Data Safety Monitoring Board has been established to assess the progress of the trial, particularly safety endpoints.

## Discussion

This clinical trial will build on previous RCTs and systematic reviews on QoL improvements using ginseng for COPD [17,22,23]. It has been designed by a multi-disciplinary and international collaborative team and will undertake a novel approached in integrating rigorous, RCT methodologies and theory that guides appropriate use of Chinese medicine for translation into clinical practice.

The trial will provide critical clinical data for effectiveness and safety of Panax ginseng C.A Meyer root extract for the improvement of QoL and pulmonary parameters such as FEV_1 _and FVC ratio. Findings from this study may lead to new therapeutic development for a range of chronic inflammatory diseases, particularly chronic respiratory diseases.

## Publication and reporting date

Late 2012 or early 2013.

## List of abbreviations

cAMP: cyclic adenosine monophosphate; CAT :COPD assessment test; COPD: chronic obstructive pulmonary disease; CTN: clinical trial notification; FEV_1_: forced expiratory volume in 1 second; FVC: forced vital capacity; GMP: good manufacturing practice; GOLD: global initiative for chronic obstructive lung disease; HREC: human research ethics committees; ICS: inhaled corticosteroids; LOCF: last observation carried forward; NHMRC: National Health and Medical Research Council; PDE4: phosphodiesterase 4; QoL: quality of life; RCT: randomised controlled trial; RMIT: Royal Melbourne Institute of Technology; SAS: statistical analysis software; SF-36: short form health survey; SGRQ: St georges respiratory questionnaire; TGA: therapeutic goods administration.

## Competing interests

The authors declare that they have no competing interests.

## Authors' contributions

This project was initiated and developed by CX. He contributed to the design of the study and development of the protocol. JS, AZ, CW, HF, DS, CDC and FT, contributed to the development of the trial protocol. All authors read and approved the final manuscript.
